# Death Anxiety Among Peer Caregivers of Older Persons in Two US Prisons

**DOI:** 10.3390/socsci14030126

**Published:** 2025-02-21

**Authors:** Stephanie Grace Prost, Warren Stewart, Meghan A. Novisky, Mary-Louise Parkkila

**Affiliations:** 1 Raymond A. Kent School of Social Work and Family Science, University of Louisville, Louisville, KY 40292, USA; 2 School of Education, Sport and Health Sciences, University of Brighton, Brighton BN2 4AT, UK; 3 Corrections Institute, University of Cincinnati, Cincinnati, OH 45206, USA; 4 College of Social Work, Florida State University, Tallahassee, FL 32304, USA

**Keywords:** death anxiety, death, prisons, corrections, peer care, caregivers, thanatophobia, prison experiences

## Abstract

**Background:**

Death anxiety is marked by worrisome thoughts and feelings surrounding death. It can influence health care workers’ performance and increase workforce attrition, yet no study has examined death anxiety among persons who provide peer care in the correctional system.

**Methods:**

Two small samples of peer caregivers working in two US prisons were surveyed (*N* = 27). Using the 15-item Death Anxiety Scale, we first described death anxiety using descriptive statistics. We examined gender disparities using an independent sample t-test and explored the associations between death anxiety, caregiver burden, and depression using Kendalls’ Tau-b.

**Results:**

Average death anxiety for the sample was 6.30 (SD = 2.88) and while women reported greater death anxiety than men, (M = 6.82, SD = 2.77; M = 5.40, SD = 2.99, respectively), the difference was not significant (t(25) = 1.25, *p* = 0.111). Although death anxiety did not relate to care burden or depression, a significant relationship was found between care burden and depression for peer caregivers in this sample.

**Conclusions:**

This is the first study to examine death anxiety among correctional system peer caregivers. Further research with larger samples, and across multiple jurisdictions and facility types is required as is investigation of the influence of death anxiety on care outcomes.

## 1. Introduction

Death anxiety or thanatophobia relates to worrisome thoughts and feelings surrounding death, including, but not limited to, life-limiting illness, the act of dying, corpses, burial, and life after death. Within the context of care dyads, correlates of death anxiety are numerous and can include depression, somatization, and pain catastrophizing in patients, as well as burden and poor care among caregivers. While there is an increasing amount of literature on peer caregiving in prisons (Stewart and Edmond 2017), very little is known regarding death anxiety among these carers. This study offers critical insight, as it is the first to describe death anxiety among peer caregivers in prisons, examine gender disparities in caregiver death anxiety, and explore the relationships between death anxiety, depression, and care burden in this important workforce.

### 1.1. Death Anxiety in Caregivers and Their Care Recipients

Death anxiety is defined as the apprehensive response to awareness of one’s finiteness (Lehto and Stein 2009). This may include affective and cognitive responses, such as unease, discomfort, or dread, related to terminal illness, the dying process, corpses, burial, and the afterlife (Lehto and Stein 2009). Death anxiety is often discussed within the context of terror management theory (TMT); in short, death anxiety emerges when efforts to stave off mortality awareness have failed (Arrowood and Cox 2020). When the buffers related to its management falter, death anxiety percolates, and psychological well-being is threatened (Juhl and Routledge 2016). While death anxiety is considered normal and universal (Lehto and Stein 2009), maladaptive presentations, such as post-traumatic stress (Martz 2004), depression (Templer 1971), somatization (Birgit et al. 2018), and pain, may manifest without proper supports. Cultural systems, then, are charged with creating and maintaining structures—symbolic or otherwise—that assure meaning while facing the imperceptible, inevitable power of death (Lehto and Stein 2009).

Within the context of care dyads, both those providing and receiving care may experience death anxiety. Among care recipients, there is an association between an individual’s health status and an increased fear of death and dying (Deaton et al. 2009; Sullivan et al. 1998); as health worsens, so does death anxiety. Not only are psychological and physical problems linked to higher levels of death anxiety, but they are also more prominent in the aging population (Benton et al. 2007; Deaton et al. 2009; Fortner and Neimeyer 1999).

While scholars are increasingly documenting positive effects of caregiving (e.g., satisfaction, happiness, pride; Lee and Li 2022), it is often described as associated with burden, depression, and anxiety (del-Pino-Casado et al. 2021; Semenova and Stadtlander 2016; Brazil et al. 2010). Caregivers are more suspectable to functional impairment, poor health, and are at an increased risk of having mental health issues (Pinquart and Sörensen 2003; Roth et al. 2009; Sallim et al. 2015; del-Pino-Casado et al. 2021). Providing care, whether informal or formal, also limits the ability to fully engage in one’s personal and social lives (viz. buffers), including employment (Pinquart and Sörensen 2003).

In a cross-sectional survey assessing death anxiety among caregivers for patients with advanced cancer, 76.3% of caregivers reported high levels of death anxiety (Ying et al. 2024). In cultures where death is taboo, or where the death education to empower individuals with knowledge and exposure related to dying and death is scarce, caregivers are less likely to discuss death, which can increase their death anxiety (Ying et al. 2024). Scholars have reported that death anxiety is greater in caregivers who have not accepted their patient’s approaching demise, compared to those who have grown to accept the forthcoming death (Semenova and Stadtlander 2016; Neimeyer et al. 2004). The level of death anxiety experienced by caregivers is also influenced by age, work related stressors, religiosity, and external death experiences (Nia et al. 2016; Wang et al. 2024). Most critically, death anxiety can influence health care workers’ performance and increase workforce attrition (Nia et al. 2016).

Scholars have reported that age and gender are associated with death anxiety. Broadly, young adults are more preoccupied with mortality compared to older adults, and women are more likely to report death anxiety compared to men (Depaola et al. 2003; Fortner and Neimeyer 1999; Iverach et al. 2014; Russac et al. 2007). Researchers sought to assess age and gender effects in death anxiety and found both men and women experienced higher levels of death anxiety in their early twenties followed by a decrease until the age of sixty. Women, however, reported higher levels of concern regarding mortality in their early twenties, with a second wave at the age of 50 (Russac et al. 2007). Further, a faster rate of death anxiety onset was observed among older women when compared to older men. Some evidence also exists that death anxiety differs between men and women caregivers (Lester et al. 2006; López-Castedo et al. 2020; Soleimani et al. 2017). Importantly, some scholars have reported the inverse or no effects regarding gender and death anxiety (Assari and Lankarani 2016).

The specific mechanisms by which gender disparities in death anxiety manifest are unclear. Gender differences could be credited to differences in self-reporting and emotional expression, as men are less likely to discuss and report such fears compared to women (Russac et al. 2007; Eshbaugh and Henninger 2013). Some relate that women experience a second wave of death anxiety as they age given that they are more likely to be caregivers in middle and older adulthood (Russac et al. 2007). Other scholars have examined the relationship between death anxiety and psychopathology. In their review of existing scholarship, authors related a link between death anxiety and depression, anxiety, mental illness symptom severity, and use of psychopharmaceuticals and hospitalizations (Gürbüz and Yorulmaz 2024). And because mental illness—specifically depression and anxiety—is overrepresented among women (Eaton et al. 2012; Mental Health Foundation 2017), women caregivers may thus be more likely to report thanatophobia.

### 1.2. The Role of the Peer Caregiver in US Prisons

Older adults constitute a large and growing number of people incarcerated in US prisons. Approximately 16 percent of the state prison population is aged 55 or older (n = 167,365; Carson and Kluckow 2023). Recent estimates relate that persons aged 50 and older will constitute 33% of the US state prison population by 2030 (National Commission on Correctional Health Care [NCCHC] 2024). Older adults incarcerated in prisons carry a heavy health burden, driven by a variety of pre-incarceration and prison-setting factors, which contributes to enormous costs related to correctional health (National Commission on Correctional Health Care [NCCHC] 2024). As one strategy to offset such expenses, aged prisoners are provided socioemotional support and aid with activities of daily living (ADLs) from peer caregivers, or other persons who are incarcerated.

A ‘caregiver’ is defined in the Care Act (2014) as an unpaid individual who assists another person with ADLs, including eating, transferring, personal hygiene, and other essential tasks. Hermanns and Mastel-Smith (2012) add that caregiving is the process of helping another person who is unable to support themselves in a “holistic” manner inclusive of physical, mental, emotional, and social spheres. The process is facilitated by specific character traits, such as empathy, knowledge, organizational skills, and an emotional connection with the care recipient. Here, “peer caregiver” refers to anyone who is incarcerated, who is also providing the main sources of care for other, vulnerable prisoners.

While the exact number of peer caregivers in the US is unknown, 44 of 45 responding state departments of corrections reported the use of palliative or end-of-life care within at least one prison in 2011 (Chari et al. 2016) and in 2016, 33 program representatives related the use of hospice or palliative care in their departmental system. Of these 33 respondents, nearly all (n = 31) indicated the use of peer caregivers within their programs (Prost et al. 2020). Authors of a review regarding end-of-life care in prisons relate that the training for peer caregivers is widely variable (Wion and Loeb 2016), with the estimates for duration ranging from ten hours to four weeks. The content contained in the trainings is similarly variable, though common topics include infection control and universal precautions, ADLs, and the philosophy of end-of-life care. Other scholars have relayed that training topics may include grief and bereavement, stress management, and confidentiality (Prost et al. 2020).

Peer caregiving in prison settings has been associated with a variety of pains and gains. For example, scholars have related that peer caregivers may experience a positive, personal transformation by supporting others in these spaces (Cloyes et al. 2014). Thus, while not developed for such purposes, peer caregiving may offer rehabilitative benefits. Others have reported that peer caregivers may receive verbal praise from other persons incarcerated and the correctional staff and many obtain intrapersonal meaning from their work supporting those with life-limiting illnesses (Stewart 2022). Some studies have relayed that material goods may also be provided to peer caregivers for their efforts (Prost et al. 2020).

Though less often described in the literature, the costs associated with peer caregiving in prison are many and distinct from caregiver experiences in the community. While several factors may drive these distinctions, it is the unique relationship between peer caregivers and their care recipients in prisons that is perhaps the most meaningful. The role filled by peer caregivers has been described as different from both professional support (viz. paid health care staff) and kinship care. Instead, peer caregivers care in ways that are both formal and familial, with previous scholars noting that caregivers report that care recipients become like family (Cloyes et al. 2014; Stewart 2022). All the while, the care dyad within carceral confines is marked by punition and power (Stewart et al. n.d.) which can give way to shared negativity (Bosson et al. 2006), as the peer caregiver and care recipient grow in intimacy based on a collective stance against the villains of the prison ecology (e.g., correctional staff, administrators, profiteers). While true among the community caregivers as well, peer caregivers come into regular contact with older adults living with chronic pain, frailty and mental distress, and according to Morse et al. (1998), these sounds, smells, and sights can assault the caregiver’s senses, leaving lasting psycho-emotional effects. Furthermore, loss pervades the incarceration experience (Hendry 2009), and feelings of mourning are likely elevated among caregivers, as the bereavement supports necessary for managing loss are limited (Prost et al. 2020). While little is known of death anxiety among peer caregivers in this space, some scholars have described death anxiety among persons incarcerated in prisons.

### 1.3. Death Anxiety in the Prison Setting

Death anxiety has historically been studied among the younger populations in comparison to those aging in carceral settings (Mullins and Lopez 1982). Some research exploring the concerns regarding dying in prison has also demonstrated that death anxiety is higher among aging incarcerated individuals compared to those in the community (Aday 2006). One study examining the effects of health and penal harm on aging females in carceral settings found that their anxiety regarding death was influenced by their perception of access to adequate health care (Deaton et al. 2009).

Anxiety and concerns related to death constitute a broad collection of issues—natural death, suicide, pre-care/after-care, and death row. According to Sim (2023), death troubles prisoners in two ways; first, the possibility of losing or grieving for a relative while serving their sentence, second, concerns about the invisibility of deaths that occur in prison. The influences on the prisoners’ views on death, dying, and death anxiety within carceral spaces may also include acute and chronic health problems, the effects of aging, mental distress, and social isolation (Aday 2006; Deaton et al. 2009; Novisky et al. 2022; Rodriguez 2013). Aday (2006) further suggests the harshness of prison life, and mistrustful and often violent relationships increase the frequency and concern of the incarcerated individuals’ thoughts of death. Worries about dying include a loss of dignity, shame and disappointment relating to failure in life fulfilment (Aday 2006; Deaton et al. 2009). Jervis (2018) reports that grief is heightened when people who are incarcerated are left to mourn alone, experiences exacerbated by disenfranchisement, or loss which is unacknowledged (Novisky et al. 2022). Therefore, attitudes towards death held by the prisoners’ mirror those of older adults in the community, but are intensified by their isolation, life experiences, and other unique facets of the carceral environment.

### 1.4. The Current Study

Driven by the potential consequences of death anxiety among both patients and caregivers, we aimed to explore death anxiety among male and female peer caregivers in two US prisons. Using secondary data derived from two phases of the Older Adults in Kentucky State Prisons (OAK) Study (2020–2022), we sought to answer the following research questions: (1) how can death anxiety be described among peer caregivers in prison using the Templer Death Anxiety Scale? (2) how does death anxiety manifest differently between genders among peer caregivers in prison? and (3) how does death anxiety relate to caregiver burden and depression among peer caregivers in prison? We anticipated that peer caregivers would experience death anxiety due to the existing scholarship relating such worries among persons who are incarcerated, and those in community caregiving roles. Further, we hypothesized that women would have greater death anxiety than men, as some evidence relates association between death anxiety and psychopathology and due to increased mental health problems among women compared to men. And because of the links between pre-existing mental illness, stress, and death anxiety, we further anticipated that as depression and caregiver burden increased, death anxiety would also increase.

## 2. Methods

A secondary data analysis of cross-sectional data was used to answer the study research questions. Data were drawn from two related efforts, with the primary data collected in 2020 (n = 21) and 2022 (n = 6). Caregivers were eligible for participation if they were above the age of 18, not in disciplinary segregation or on suicide watch, able to speak and read English, and providing some sort of care—paid or unpaid—for older adults (e.g., those 50+ years of age) incarcerated in prison.

For our 2020 respondents, caregivers completed questionnaires via face-to-face interviews across two prisons. Questionnaires included measures related to caregiver experiences prior to and during incarceration, health, life quality, care burden, and death anxiety. Due to the COVID-19 pandemic and institutional closures, our initial data collection efforts were stopped, and revised protocols were submitted and approved by the external funder, prison, and the university’s human subjects’ research institutional review board (IRB). We then conducted a mail questionnaire for the 2022 respondents, with fewer queries to reduce burden. We mailed packets into two facilities, inclusive of a personalized, introductory letter, a step-by-step guide and checklist, university and Department of Corrections (DOC) consent documents, and the questionnaire. Papers were bound yet staple free, and addresses and postage were printed. Respondents completed the questionnaire at their discretion and the principal investigator (Prost) retrieved the responses from a locked box placed in a secure location (e.g., prison library). The hope was that doing so would allow the respondents to provide answers with less fear of staff review, as all outgoing mail is subject to search.

For the 2020 respondents, we approached all caregivers identified by institutional administrators as having met the study inclusion criteria to consent (viz. population census; N = 22). Twenty-one agreed to participate, representing a 95.5% response rate. For the 2022 respondents, we mailed surveys to all 38 caregivers identified by institutional administrators who met the study inclusion criteria. We received 6 completed surveys, representing a 15.8% response rate.

## 3. Analyses

Research questions 1 and 2 were answered using data from the 2020 and 2022 respondents. Research question 3 was answered using data from the 2020 respondents. We used descriptive statistics including frequencies, proportions, and means to describe death anxiety among peer caregivers. We leveraged a Mann–Whitney U test to examine 2020 and 2022 sample responses for statistically significant differences and then examined gender disparities in death anxiety using an independent sample *t*-test. Finally, we explored the associations among death anxiety, depression, and caregiver burden using Kendalls’ Tau-b, a nonparametric correlation coefficient that provides a measure of strength and direction among data not normally distributed.

## 4. Measures

The sociodemographic characteristics of peer caregivers included in the current study were as follows: gender, primary race, highest completed level of education, and marital status. Additional caregiver characteristics were included for the 2020 interviewees, including caregiver status, experience caregiving prior to prison, and whether items or other benefits were received for providing care. We also asked those caregivers to rate how caregiving has influenced them and has changed their outlook on the future, each on a scale from 0 to 10, with 0 relating to ‘very negatively’ and 10 relating to ‘very positively’.

Death anxiety was captured using the 15-item Death Anxiety Scale (DAS; Templer 1971). Considered the most common self-report measure of death anxiety, the DAS has been translated into 26 languages, is largely atheoretical (Lehto and Stein 2009), and has been reported as possessing ample evidence of validity and reliability (Zuccala et al. 2022). Some scholars have explored the underlying structure of the measure, and have put forth a two-factor framework, including psychological or internal factors and life experience or external factors (Tomer 1992). Items in the DAS are constructed to represent either true (n = 9) or false (n = 6) responses. Examples include “the thought of death never bothers me” and “the thought of death seldom enters my mind” (keyed false) and “I fear dying a painful death”, and “I am really scared of having a heart attack” (keyed true). For the current study, caregiver scores were computed by obtaining a sum of responses (possible range: 0–15; Iverach et al. 2014). Here, 0 relates to no death anxiety and 15 relates to the greatest death anxiety.

Depression was assessed using the eight-item version of the Patient Health Questionnaire (PHQ-8; Kroenke et al. 2001). The measure includes items that reflect the criteria essential to diagnosing a depressive episode per the DSM-5. The measure also provides a level of severity (Kroenke et al. 2001). Caregivers are asked to consider the previous two weeks and to indicate the frequency at which they experienced the criteria. Response options (4) ranged from zero (not at all) to three (nearly every day), and the caregiver scores were computed by obtaining the sum of selected responses (possible range: 0 to 24), where higher scores equate to greater depression. PHQ-8 scores greater than 10 indicate the presence of depression (Kroenke and Spitzer 2002).

Caregiver burden was measured using the Zarit Burden Inventory (ZBI; Zarit et al. 1980). A 21-item version of the measure was used with response options (5) ranging from 0 (never) to 4 (nearly always). Caregivers were asked to choose the response that best describes how often they feel a certain way regarding their primary caree, the person for whom they provide the most care. Instructions also clarified that there are no ‘right’ or ‘wrong’ answers. Example items include “Do you feel embarrassed over your caree’s behavior?” “Are you afraid what the future holds for your caree?” and “Do you feel you should be doing more for your caree?” Caregiver scores were computed by obtaining the sum of selected responses (possible range: 0–84). Higher scores reflect a greater level of care burden.

Sample Demographics. The sample was mostly women (n = 17; 2020 = 13, 2022 = 4), and primarily white (n = 18, 66.7%), though African American (n = 6, 22.2%) and American Indian or Alaskan Native caregivers (n = 1, 3.7%) were also represented. Caregivers most often completed high school (n = 21, 77.8%) and were divorced or separated (n = 11, 40.7%) or never married (n = 9, 33.3%). The mean age of caregivers was 42.37 (SD = 8.81). Of those queried (n = 21), peer caregivers reported having provided care in prison for between 4 months and 8 years and for 2 to 12 hours per day. Seventy-six percent reported having experience as a caregiver prior to prison (n = 16). Most caregivers indicated they were caring in a paid, formalized role (n = 13); the remaining indicated they volunteer as caregivers (n = 8).

## 5. Results

Among the 2020 interviewees, 62% reported receiving money/pay for their efforts (n = 13), 24% related the receipt of food or drinks (n = 5), 24% reported having been provided belongings (n = 5), 48% reported having been given esteem by peers (n = 10), and 91% reported having received appreciation from staff (n = 19). Finally, over 70% of carers surveyed reported that caregiving has influenced them ‘very positively’ (viz. rating of 10; n = 15). Moreover, 48% of those asked about the influence of caregiving on their outlook on the future rated the experience ‘very positively’ (n = 10); only one caregiver reported caregiving as having changed their outlook ‘slightly negatively’ (viz. rating of 5).

The mean death anxiety score for the full sample was 6.30 (SD = 2.88). Regarding individual death anxiety items, carers reported thinking “about how short life really is” most often (n = 20), followed by ‘I fear dying a painful death’ (n = 15), and ‘I am really scared of having a heart attack’ (n = 13). The fewest number of peer caregivers indicated ‘I am very much afraid to die’ (n = 6). Responses to each item in the DAS can be found in Table A1. The mean depression score for the sample was 5.86 (SD = 4.14; observed range 0–15) and the mean burden score was 17.70 (SD = 9.43; observed range: 7–44).

Results from the Mann–Whitney U test relate that significant differences in death anxiety did not emerge between 2020 and 2022 administrations (*U* = 50.5; *z* = 0.699, *p* = 0.484). And while women scored higher on the DAS than men (M = 6.82, SD = 2.77; M = 5.40, SD = 2.99, respectively), the total score for death anxiety did not differ significantly between male and female caregivers (t(25) = 1.25, *p* = 0.111). Further, death anxiety did not relate to depression or caregiver burden among the 2020 interviewees. However, the relationship between caregiver burden and depression was significant (τb = 0.396, *p* = 0.018, n = 20; Table A2).

### 5.1. Discussion

We sought to describe death anxiety ratings among peer caregivers in prison, examine differences in death anxiety between male and female prison peer caregivers, and explore how death anxiety related to caregiver burden and depression among prison peer caregivers. We anticipated that peer caregivers in prison would experience death anxiety, that women would have greater death anxiety than men, and that death anxiety would relate positively to depression and caregiver burden. Overall, the findings affirm our hypotheses only partially.

As anticipated, peer caregivers did report death anxiety. Peer caregivers’ average death anxiety score hovered at 6.30 on the DAS (maximum score of 15). While some have argued that cross-cultural comparisons of death anxiety are problematic (Beshai 2008) and norms for the DAS are rarely cataloged (Stevens et al. 1980), we offer some context for these values. Norms for the DAS published in 1980 relate that the average score in a sample of 295 adults aged 16–83 was 6.89 (SD = 3.20). Further, the average score for 71 persons aged 40 to 59 was 6.85 (SD = 2.77) and 5.74 (SD = 2.95) for 68 persons aged 60 to 83. Thus, the average score reported in the current study may be lower than established norms in the general, non-incarcerated population. However, Aday (2006) used the DAS in a study of aging persons incarcerated in the Mississippi Department of Corrections. The average DAS score for that sample was 5.02 (n = 102). Deaton et al. (2009) later report a mean score of 6.40 on the DAS with a sample of women (n = 327) aged 50 or older incarcerated in five Southern states. However, higher levels have been reported among some community caregivers; for example, the average score for death anxiety among those caring for persons with advanced cancer in China was 7.92 using the DAS (Ying et al. 2024). Several contextual factors could explain the disparities between these studies, such as localized cultural differences, distinctions in the quality of teamworking or support from the wider multidisciplinary team, or differences in training. The differing results could equally reflect methodological differences such as sample sizes. It is also possible that the context of the COVID-19 pandemic played a role in elevating death anxiety among the 2022 subsample.

The greatest number of peer caregivers reported death anxiety related to ‘how short life really is’. At face value, the high score in the item suggests ominous connotations; however, the interpretation of this finding is not necessarily a negative complexion of their experience. As the peer caregivers observe the physical changes and functional decline of those they are caring for, it is possible that caregivers begin to feel a heightened sense of their own brevity. Exposure to critically unwell and dying peers could equally inspire thoughts relating to seeing beyond their immediate situations, and making the most of a restricted life, and therefore could be interpreted as the development of a new insight or positive existential reflections. For example, other authors have alluded to post-traumatic growth as a gain associated with peer caregiving in prisons (Depner et al. 2017). Toch (2000) suggests such altruistic activities and accomplishments can result in cognitive restructuring and increments in self-esteem. When caregivers are face-to-face with functional decline or terminal illness, however, they might begin to feel a loss of control (Russac et al. 2007), reflecting on their personal goals, dreams, and aspirations as realities they will not experience and thereby, the finality of life. The knowledge of one’s limitations has the capacity to increase anxiety (Pyszczynski 2019). Like in the results reported by Ying et al. (2024), peer caregivers also reported thinking about dying a painful death and being afraid about having a heart attack. These findings are consistent with qualitative accounts of older incarcerated individuals fearing such outcomes, based on their perceptions of the medical response times and the witnessed suffering of their incarcerated peers (Novisky 2018; Novisky et al. 2022).

In contrast, peer caregivers were least likely to affirm ‘I am very much afraid to die’. This may indicate a familiarity with dying and death and, thereby, a reduced fear regarding this facet of mortality awareness. Other scholars have reported that older persons in prisons are faced regularly with death and dying (Novisky et al. 2022). Death can be accepted through the process of familiarization (Wysokiński et al. 2019), such as being a caregiver, witnessing serious illness or even the death of their peers. This finding also parallels work by Ying et al. (2024), who relate that caregivers become more aware of mortality when working with those near death. However, it is possible that this finding reflects an attitude of avoidance towards death (viz. death denial; Ying et al. 2024), leading to decreased affirmation regarding the phrase ‘I am very much afraid to die’. Lastly, a peer caregivers’ spirituality might contribute to a decreased fear of dying. Wysokiński et al. (2019) note that religion could facilitate the acceptance of death. For those who hold hope in an afterlife, the fear of death is not as prominent (Neimeyer et al. 2004). These are important areas of exploration for future scholars.

This low scoring response could also reflect the prevailing prisoner culture, as it is known that incarcerated individuals and prison staff operate according to well defined codes, nested in masculinity contest cultures (Berdahl et al. 2018), which are distinct from cultures found in community health care or hospice settings. Vulnerability is perceived as a sign of weakness and therefore suppressed as a feature of their external identity. Therefore, a low score on this item could reflect an internalized ‘stiff upper lip’ approach to dealing with emotional issues, instrumental thinking, or the sublimation of unwanted anxiety via the use of avoidance or dark humor.

While significant differences did not emerge between male and female caregivers as anticipated, women did report higher levels of death anxiety than men in this sample. This may be due to a generally increased mental distress among women when compared to men in these settings. However, it is also possible that women experienced empathy and compathy toward their care recipients in ways distinct from their counterparts. The phenomenon of ‘compathy’ is an involuntary physical reaction to the care recipients’ distress, which entails ‘the acquisition of physical distress and/or physiological symptoms (including pain) by an apparently healthy individual who perceives another’s suffering or who privately contemplates the experience of another’s illness’ (Morse et al. 1998, p. 55). The effects of the compathy phenomenon can manifest in two ways: it can either motivate caregivers to provide care, or can cause them to ‘shield’ or ‘steel’ themselves–in other words defend against the anxiety caused by distress, for example, by objectifying patients or ignoring humanistic elements of their role (Lyth 1988). The current study did not examine shielding or steeling responses, but 2020 respondents were asked to report how caregiving has influenced them and their outlook on the future. As more than 70% of caregivers reported the experience influenced them ‘very positively’, it is possible that this may represent motivation rather than shielding or steeling responses.

While the mean depression score for the sample meets the criteria for mild symptom severity, based on the instrument developer guidance (Kroenke et al. 2001), other studies of depression in caregivers of persons with chronic illness, using the PHQ-8, have related lower scores (Hwang et al. 2012; Pressler et al. 2009). And while the mean burden score for our sample was 17.70, this is lower than the average ZBI scores reported among caregivers of persons with Alzheimer’s Disease (M = 25.98, Froelich et al. 2021), Parkinson’s Disease (M = 28.3, Hagell et al. 2017), and cancer (M = 54.75, Moghaddam et al. 2023).

Finally, we did not find a significant relationship between death anxiety, care burden, and depression. There could be several reasons for this. As noted in the literature, caregivers can extract some intrinsic gains from involvement in supportive activities aligned to changes in life orientations or cognitive schemas, such as redemption narratives (McAdams et al. 2001), wounded healer narratives (LeBel et al. 2015), and helper narrative principle (Riessman 1965). They can perceive a higher meaning in the work for spiritual or religious reasons or, particularly among male participants, they might actively avoid the acknowledgement or reporting of dissonance or emotional distress, which could challenge self-perceptions of masculine status.

### 5.2. Limitations

The principal limitations of the current study are the small sample size across two time points, and the inclusion of participants from only one state prison system. Future scholars are thus encouraged to explore death anxiety and related constructs among larger and potentially random samples of peer caregivers across multiple jurisdictions to allow for the detection of a smaller effect. And as many differences likely exist between institutional settings, additional measures related to training, support, and staffing are worthy of inclusion in multivariate models to account for variation in death anxiety scores and differences between men and women caregivers. Likewise, prison social support networks could vary by gender, which may serve as an important factor in death anxiety. The inclusion of specific tasks completed by caregivers may also prove an important advancement. Qualitative approaches are likely indicated to develop theory, as well. The development and validation of caregiver burden measures, that more adequately reflect the unique formal–familial role of peer caregivers, is also encouraged. An additional consideration for future researchers is the use of other measures of death anxiety, such as the 20-item Death Anxiety Inventory (Gómez et al. 2007) or perhaps the 42-item Multidimensional Fear of Death Scale (MFODS; Hoelter 1979; Walkey 1982). The use of measures with additional subscales, however, must be weighed against respondent and institutional burden (Dillman et al. 2014). Despite these limits, we provide a foundation for future scholars and practitioners regarding the presence of death anxiety in peer caregivers. Given how little is known about death anxiety among prison peer carers in the literature, as well as how difficult it can be to sample prison peer carers, the findings presented in this paper are important to disseminate.

### 5.3. Implications for Research and Practice

Researchers are encouraged to continue assessing death anxiety among peer caregivers and other constituencies (e.g., correctional officers, health care providers) within carceral settings. Researchers are also encouraged to assess the role of TMT in death anxiety among peer caregivers. TMT theorists posit that thoughts surrounding mortality can overwhelm individuals, contributing to death anxiety without the application of various buffers. For example, examining buffers or protective factors at the cultural, relational, and intrapsychic levels would offer insights regarding the variable strength of defenses against mortality awareness and thereby, opportunities for intervention to reduce death anxiety among peer caregivers, and enhance care outcomes for their care recipients. Relatedly, it may also prove an important line of inquiry to examine the influence of benefits (viz. intrapsychic buffers) and perceived pains of caregiving, on stress, burden, and death anxiety.

In terms of practice, various interventions have been utilized to mitigate death anxiety, including Cognitive Behavioral Therapy (CBT) and death education programs (Furer and Walker 2008; Menzies et al. 2018; Saki et al. 2022). A recent trial seeking to explore the safety and benefits of an online CBT-based treatment for death anxiety showed a reduction in at least one aspect of death anxiety after seven modules (Menzies et al. 2023). This treatment addresses unhelpful thinking patterns, challenges beliefs about death, promotes exposure to death concepts, and encourages individuals to create their own value system (Menzies et al. 2018, 2023). The online format is particularly valuable, as similar strategies can be adapted to provide virtual interventions for death anxiety for peer caregivers in prison settings.

Death education interventions–especially those with experiential components–may reduce death anxiety (Menzies et al. 2018; Kim 2015). Research surrounding death education groups is limited, but serves as a launching point for exposing peer caregivers to common concepts regarding the processes of death and to adaptive coping skills for navigating grief. Research on treatment and interventions for death anxiety in a clinical context are limited. Given the available data, CBT appears to be the most effective in addressing the multifaceted nature of death anxiety (Menzies et al. 2018).

Stewart (2018) notes the use of an educational workshop entitled ‘working with loss’, driven by peer caregiver feedback. And Prost et al. (2020) relate the need for bereavement support for peer caregivers in prison hospice settings. Death education groups with peer caregivers could be utilized to provide psychoeducation and coping skills, while allowing peer caregivers to discuss death anxiety as a shared experience. Opportunities such as these, allowing for the debrief and off-loading of troublesome experiences, provide both the release to those speaking and insights to those listening. With a guided reflection on the application of these and similar topics, peer caregivers may further develop meta-competencies in self-efficacy and decision-making, which would translate to improved care and patient outcomes. Meeting in a group setting and sharing such experiences is also anticipated to reduce attrition and support longevity among peer caregivers (Stewart 2018). We would further encourage for loss-related supports to be available to all incarcerated persons due to the pervasiveness of grief in these spaces.

Regardless of the specific approach to mitigating death anxiety, acknowledgement and intervention by prison administrators and staff is anticipated to benefit caregivers and their charges. While peer caregiving is unlikely to translate to real-world job opportunities, due to felony exclusions and stigma in health care (Mach 2025), downstream impacts on the patients receiving care and the system, more broadly, warrant timely and targeted support.

## Data Availability

The original contributions presented in this study are included in the article. Further inquiries can be directed to the corresponding author.

## Appendix A

**Table A1.** Death anxiety among peer caregivers in prison (N = 27).

	n	%
I am very much afraid to die	6	22.2
The thought of death seldom enters my mind	13	48.1
It doesn’t make me nervous when people talk about death	18	66.7
I dread to think about having to have an operation	11	40.7
I am not at all afraid to die	18	66.7
I am not particularly afraid of getting cancer	10	37
The thought of death never bothers me	10	37
I am often distressed by the way time flies so very rapidly	8	29.6
I fear dying a painful death	15	55.6
The subject of life after death troubles me greatly	4	14.8
I am really scared of having a heart attack	13	48.1
I often think about how short life really is	20	74.1
I shudder when I hear people talking about World War 3	2	7.4
The sight of a dead body is horrifying to me	7	25.9
I feel the future holds nothing to fear	9	33.3

**Table A2.** Relationships between death anxiety, depression, and caregiver burden among peer caregivers in prison (n = 21).

	Caregiver Burden	Death Anxiety
Death anxiety	0.072	
Depression	0.396 *	0.251

Note:

* significant *p* < 0.05.

## References

[R1] Aday Ronald H. 2006. Aging prisoners’ concerns toward dying in prison. OMEGA-Journal of Death and Dying 52: 199–216.

[R2] Arrowood Robert B., and Cox Cathy R.. 2020. Terror management theory: A practical review of research and application. Brill Research Perspectives in Religion and Psychology 2: 1–83.

[R3] Assari Shervin, and Lankarani Maryam M.. 2016. Race and gender differences in correlates of death anxiety among elderly in the United States. Iranian Journal of Psychiatry and Behavioral Sciences 10: e2024. 27803717 10.17795/ijpbs-2024 PMC5088440

[R4] Benton Joshua P., Christopher Andrew N., and Walter Michael I.. 2007. Death anxiety as a function of aging anxiety. Death Studies 31: 337–50. 17378111 10.1080/07481180601187100

[R5] Berdahl Jennifer L., Cooper Marianne, Glick Peter, Livingston Robert W., and Williams Joan C.. 2018. Work as a Masculinity Contest. Journal of Social Issues 74: 422–48.

[R6] Beshai James A. 2008. Are cross-cultural comparisons of norms on death anxiety valid? OMEGA-Journal of Death and Dying 57: 299–313. 10.2190/OM.57.3.e 18837176

[R7] Birgit Maes, Tak Laura M., Rosmalen Judith G., and Oude Voshaar Richard C.. 2018. Death anxiety and its association with hypochondriasis and medically unexplained symptoms: A systematic review. Journal of Psychosomatic Research 115: 58–65. 30470318 10.1016/j.jpsychores.2018.10.002

[R8] Bosson Jennifer K., Johnson Amber B., Niederhoffer Kate, and Swann William B. Jr. 2006. Interpersonal chemistry through negativity: Bonding by sharing negative attitudes about others. Personal Relationships 13: 135–50.

[R9] Brazil Kevin, Bainbridge Daryl, and Rodriguez Carmen. 2010. The stress process in palliative cancer care: A qualitative study on informal caregiving and its implication for the delivery of care. American Journal of Hospice and Palliative Medicine 27: 111–16. 20008823 10.1177/1049909109350176

[R10] Care Act. 2014. UK Government legislation. Available online: https://www.legislation.gov.uk/ukpga/2014/23/contents (accessed on 5 July 2024).

[R11] Carson Elizabeth A., and Kluckow Rachel. 2023. Correctional Populations in the United States, 2021—Statistical Tables. Washington, DC: Bureau of Justice Statistics.

[R12] Chari Karishma A., Simon Anne E., DeFrances Carol J., and Maruschak Laura. 2016. National survey of prison health care: Selected findings. National Health Statistics Reports 2016: 1–23. 27482922

[R13] Cloyes Kristin G., Rosenkranz Susan J., Wold Debra, Berry Patricia H., and Supiano Katherine P.. 2014. To be truly alive: Motivation among prison inmate hospice volunteers and the transformative process of end-of-life peer care service. American Journal of Hospice and Palliative Medicine 31: 735–48. 24071627 10.1177/1049909113506035

[R14] Deaton Debra, Aday Ronald H., and Wahidin Azrini. 2009. The effect of health and penal harm on aging female prisoners’ views of dying in prison. OMEGA-Journal of Death and Dying 60: 51–70. 10.2190/om.60.1.c 20039531

[R15] del-Pino-Casado Rafael, Priego-Cubero Eva, López-Martínez Catalina, and Orgeta Vasiliki. 2021. Subjective caregiver burden and anxiety in informal caregivers: A systematic review and meta-analysis. PLoS ONE 16: e0247143. 33647035 10.1371/journal.pone.0247143 PMC7920375

[R16] Depaola Stephen J., Griffin Melinda, Young John R., and Neimeyer Robert A.. 2003. Death anxiety and attitudes toward the elderly among older adults: The role of gender and ethnicity. Death Studies 27: 335–54. 12749378 10.1080/07481180302904

[R17] Depner Rachel M., Grant Peter C., Byrwa David J., Breier Jamie M., Lodi-Smith Jennifer, Kerr Charles W., and Luczkiewicz Debra L.. 2017. A consensual qualitative research analysis of the experience of inmate hospice caregivers: Posttraumatic growth while incarcerated. Death Studies 41: 199–210. 27874320 10.1080/07481187.2016.1237591

[R18] Dillman Don A., Smyth Jolene D., and Christian Leah Melani. 2014. Internet, Phone, Mail, and Mixed-Mode Surveys: The Tailored Design Method. Hoboken: John Wiley & Sons.

[R19] Eaton Nicholas R., Keyes Katherine M., Krueger Robert F., Balsis Steve, Skodol Andrew E., Markon Kristian E., and Hasin Deborah S.. 2012. An invariant dimensional liability model of gender differences in mental disorder prevalence: Evidence from a national sample. Journal of Abnormal Psychology 121: 282. 21842958 10.1037/a0024780 PMC3402021

[R20] Eshbaugh Elaine, and Henninger William. 2013. Potential Mediators of the Relationship Between Gender and Death Anxiety. Individual Differences Research, 11.

[R21] Fortner Barry V., and Neimeyer Robert A.. 1999. Death anxiety in older adults: A quantitative review. Death Studies 23: 387–411. 10558505 10.1080/074811899200920

[R22] Froelich Lise, Lladó Anna, Khandker Rezaul K., Pedrós Marta, Black Christine M., Sánchez Díaz Eva J., and Ambegaonkar Baishali M.. 2021. Quality of life and caregiver burden of Alzheimer’s Disease among community dwelling patients in Europe: Variation by disease severity and progression. Journal of Alzheimer’s Disease Reports 5: 791–804. 10.3233/ADR-210025 PMC8609484 34870105

[R23] Furer Patricia, and Walker John R.. 2008. Death anxiety: A cognitive-behavioral approach. Journal of Cognitive Psychotherapy 22: 167–82.

[R24] Gómez Juan, Hidalgo María D., and Tomás-Sábado Joaquín. 2007. Using polytomous item response models to assess death anxiety. Nursing Research 56: 89–96. 17356439 10.1097/01.NNR.0000263966.24125.92

[R25] Gürbüz Ahmet, and Yorulmaz Orhan. 2024. Death Anxiety in Psychopathology: A Systematic Review. Psikiyatride Güncel Yaklaşımlar 16: 159–74.

[R26] Hagell Peter, Alvariza Anette, Westergren Anna, and Årestedt Kristofer. 2017. Assessment of burden among family caregivers of people with Parkinson’s disease using the zarit burden interview. Journal of Pain and Symptom Management 53: 272–78. 27810571 10.1016/j.jpainsymman.2016.09.007

[R27] Hendry Caroline. 2009. Incarceration and the tasks of grief: A narrative review. Journal of Advanced Nursing 65: 270–78. 19191932 10.1111/j.1365-2648.2008.04890.x

[R28] Hermanns Melinda, and Mastel-Smith Beth. 2012. Caregiving: A qualitative concept analysis. Qualitative Report 17: 75.

[R29] Hoelter Jon W. 1979. Multidimensional treatment of fear of death. Journal of Consulting and Clinical Psychology 47: 996. 512155 10.1037//0022-006x.47.5.996

[R30] Hwang Beomjong, Howie-Esquivel Jill, Fleischmann Kirsten E., Stotts Nancy A., and Dracup Kathleen. 2012. Family caregiving in pulmonary arterial hypertension. Heart & Lung 41: 26–34. 21592575 10.1016/j.hrtlng.2011.03.002

[R31] Iverach Lisa, Menzies Ross G., and Menzies Rachel E.. 2014. Death anxiety and its role in psychopathology: Reviewing the status of a transdiagnostic construct. Clinical Psychology Review 34: 580–93. 25306232 10.1016/j.cpr.2014.09.002

[R32] Jervis Jane. 2018. Mourning in custody: Dealing with sudden death. In Loss, Dying and Bereavement in the Criminal Justice System. Milton Park: Routledge, pp. 160–67.

[R33] Juhl Jacob, and Routledge Clay. 2016. Putting the terror in terror management theory: Evidence that the awareness of death does cause anxiety and undermine psychological well-being. Current Directions in Psychological Science 25: 99–103.

[R34] Kim Sun H. 2015. A meta analysis of effectiveness of death education. The Korean Journal of Hospice and Palliative Care 18: 196–207.

[R35] Kroenke Kurt, Spitzer Robert L., and Williams Janet B. W.. 2001. Validity of a brief depression severity measure. Journal of General Internal Medicine 16: 606–13. Available online: https://onlinelibrary.wiley.com/doi/pdf/10.1046/j.1525-1497.2001.016009606.x (accessed on 5 February 2025). 11556941 10.1046/j.1525-1497.2001.016009606.x PMC1495268

[R36] Kroenke Kurt, and Spitzer Robert L. 2002. The PHQ-9: A new depression diagnostic and severity measure. Psychiatric Annals 32: 509–15.

[R37] LeBel Thomas P., Richie Matt, and Maruna Shadd. 2015. Helping others as a response to reconcile a criminal past: The role of the wounded healer in prisoner reentry programs. Criminal Justice and Behavior 42: 108–120.

[R38] Lee Yeonjung, and Li Lydia. 2022. Evaluating the positive experience of caregiving: A systematic review of the positive aspects of caregiving scale. The Gerontologist 62: e493–e507. 34216215 10.1093/geront/gnab092

[R39] Lehto Rebecca, and Stein Karen F.. 2009. Death anxiety: An analysis of an evolving concept. Research and Theory for Nursing Practice 23: 23–41. 19418886 10.1891/1541-6577.23.1.23

[R40] Lester David, Templer Donald I., and Abdel-Khalek Ahmed M.. 2006. Cross-cultural comparisons of Death Anxiety. OMEGA 54: 255–60. 17847957 10.2190/w644-8645-6685-358v

[R41] López-Castedo Alicia, González-Rodríguez Raquel, and Perez Ricardo Vazquez. 2020. Psychometric properties of the Death Anxiety Scale in patients with ischemic cardiomyopathy. Revista Española de Salud Pública 93: e201910079. PMC11583031 31576814

[R42] Lyth Isabel Menzies. 1988. Containing Anxiety in Institutions: Selected Essays. London: Free Association Books, vol. 1.

[R43] Mach John. 2025. From Prison Bars to Care Bars—Can Felons Become Caregivers? Available online: https://blog.medicsignal.com/can-felons-become-caregivers/ (accessed on 5 February 2025).

[R44] Martz Erin. 2004. Death anxiety as a predictor of posttraumatic stress levels among individuals with spinal cord injuries. Death Studies 28: 1–17. 14969275 10.1080/07481180490249201

[R45] McAdams Dan P., Reynolds Jeffrey, Lewis Martha, Patten Allison H., and Bowman Phillip J.. 2001. When Bad Things Turn Good and Good Things Turn Bad: Sequences of Redemption and Contamination in Life Narrative and Their Relation to Psychosocial Adaption in Midlife Adults and in Students. Personality and Social Psychology Bulletin 27: 474–85.

[R46] Mental Health Foundation. 2017. Men and Women: Statistics. Available online: https://www.mentalhealth.org.uk/explore-mental-health/statistics/men-women-statistics (accessed on 5 February 2025).

[R47] Menzies Rachel E., Julien Annabel, Sharpe Louise, Menzies Ross G., Helgadóttir Fjola D., and Dar-Nimrod Ilan. 2023. Overcoming death anxiety: A phase I trial of an online CBT program in a clinical sample. Behavioural and Cognitive Psychotherapy 51: 374–79. 36961120 10.1017/S135246582300005X

[R48] Menzies Rachel E., Zuccala Matteo, Sharpe Louise, and Dar-Nimrod Ilan. 2018. The effects of psychosocial interventions on death anxiety: A meta-analysis and systematic review of randomised controlled trials. Journal of Anxiety Disorders 59: 64–73. 30308474 10.1016/j.janxdis.2018.09.004

[R49] Moghaddam Zahra, Rostami Mahdi, Zeraatchi Alireza, Bytamar Jalal Mohammadi, Saed Omid, and Zenozian Saeed. 2023. Caregiving burden, depression, and anxiety among family caregivers of patients with cancer: An investigation of patient and caregiver factors. Frontiers in Psychology 14: 1059605. 37057172 10.3389/fpsyg.2023.1059605 PMC10086361

[R50] Morse Janice M., Mitcham Carla, and van Der Steen Wim J.. 1998. Compathy or physical empathy: Implications for the caregiver relationship. Journal of Medical Humanities 19: 51–65.

[R51] Mullins Larry C., and Lopez Maria A.. 1982. Death anxiety among nursing home residents: A comparison of the young-old and the old-old. Death Education 6: 75–86. 10254759 10.1080/07481188208252117

[R52] National Commission on Correctional Health Care [NCCHC]. 2024. Care for Aging Patients in the Correctional Setting. Available online: https://www.ncchc.org/position-statements/care-for-aging-patients-in-the-correctional-setting/ (accessed on 5 February 2025). 10.1089/jchc.2024.89563.AP 39441089

[R53] Neimeyer Robert A., Wittkowski Joachim, and Moser Robert P.. 2004. Psychological research on death attitudes: An overview and evaluation. Death Studies 28: 309–40. 15129688 10.1080/07481180490432324

[R54] Nia Hamid Sharif, Lehto Rebecca H., Ebadi Abbas, and Peyrovi Hamid. 2016. Death anxiety among nurses and health care professionals: A review article. International Journal of Community Based Nursing and Midwifery 4: 2. 26793726 PMC4709813

[R55] Novisky Meghan A. 2018. Avoiding the runaround: The link between cultural health capital and health management among older prisoners. Criminology 56: 643–78.

[R56] Novisky Meghan A., Narvey Chelsea S., and Prost Stephanie Grace. 2022. Older Adults’ perspectives on death and dying in prison. Journal of Criminal Justice 83: 101930.

[R57] Pinquart Martin, and Silvia Sörensen. 2003. Differences between caregivers and noncaregivers in psychological health and physical health: A meta-analysis. Psychology and Aging 18: 250. 12825775 10.1037/0882-7974.18.2.250

[R58] Pressler Susan J., Gradus-Pizlo Irmina, Chubinski Susan D., Smith Gordon, Wheeler Sandra, Wu Jia-Rong, and Sloan Robert. 2009. Family caregiver outcomes in heart failure. American Journal of Critical Care 18: 149–59. 19255105 10.4037/ajcc2009300 PMC2651565

[R59] Prost Stephanie Grace, Holland Margaret M., Hoffmann Heath C., and Dickinson George E.. 2020. Characteristics of hospice and palliative care programs in US prisons: An update and 5-year reflection. American Journal of Hospice and Palliative Medicine 37: 514–20. 31808349 10.1177/1049909119893090

[R60] Pyszczynski Tom. 2019. A terror management theory perspective on human motivation. In The Oxford Handbook of Human Motivation. Oxford: Oxford University Press, pp. 67–88.

[R61] Riessman Frank. 1965. The ‘Helper’ Therapy Principle. Social Work 10: 27–32.

[R62] Rodriguez Anthony. 2013. Through the Looking Glass: Integrating Male Prisoners’ Perceptions and Views of Death. Doctoral dissertation, University of Alabama Libraries, Tuscaloosa, AL, USA.

[R63] Roth David L., Perkins Melissa, Wadley Virginia G., Temple Elizabeth M., and Haley William E.. 2009. Family caregiving and emotional strain: Associations with quality of life in a large national sample of middle-aged and older adults. Quality of Life Research 18: 679–88. 19421895 10.1007/s11136-009-9482-2 PMC2855243

[R64] Russac RJ, Colleen Gatliff, Mimi Reece, and Dihann Spottswood. 2007. Death anxiety across the adult years: An examination of age and gender effects. Death Studies 31: 549–61. 17726829 10.1080/07481180701356936

[R65] Saki Maryam, Khoshnood Sareh, Mohammadipour Fatemeh, Ebrahimzadeh Farzad, and Rezaei Farzin. 2022. The effect of cognitive–behavioral intervention on hope and death anxiety level in patients undergoing hemodialysis. The Journal of Mental Health Training, Education and Practice 17: 181–90.

[R66] Sallim Adnaan Bin, Sayampanathan Andrew Arjun, Cuttilan Amit, and Ho Roger Chun-Man. 2015. Prevalence of mental health disorders among caregivers of patients with Alzheimer disease. Journal of the American Medical Directors Association 16: 1034–41. 26593303 10.1016/j.jamda.2015.09.007

[R67] Semenova Vera, and Stadtlander Lisa. 2016. Death anxiety, depression, and coping in family caregivers. Journal of Social Behavioral, and Health Sciences 10: 5.

[R68] Sim Joe. 2023. Death, Trauma and Grief: The Case of the Prison. Mortality 28: 269–83.

[R69] Soleimani Mohammad Ali, Lehto Rebecca H., Negarandeh Reza, Bahrami Nasim, and Chan Yiong Huak. 2017. Death anxiety and quality of life in Iranian caregivers of patients with cancer. Cancer Nursing 40: E1–E10. 10.1097/NCC.0000000000000355 26925995

[R70] Stevens Sandra J., Cooper Patricia E., and Thomas Lawrence E.. 1980. Age norms for templer’s death anxiety scale. Psychological Reports 46: 205–6. 7367537 10.2466/pr0.1980.46.1.205

[R71] Stewart Warren. 2018. What Does the Implementation of Peer Care Training Reveal About Prisoner Engagement in Peer Care Giving? Journal of Forensic Nursing 14: 18–26. 29461380 10.1097/JFN.0000000000000183

[R72] Stewart Warren. 2022. ‘Helping Not Hurting’: Horizontal Care and Learning to Peer Care in Prison. Ethics and Social Welfare 16: 90–105.

[R73] Stewart Warren, and Edmond Nadia. 2017. Prisoner Peer Caregiving: A literature review. Nursing Standard RCNi 31: 44–51. 10.7748/ns.2017.e10468 28378680

[R74] Stewart Warren, Prost Stephanie Grace, Novisky Meghan A., Archuleta Adrian J., and Golder Seana. n.d. Subjective and inter-subjective meanings associated with peer caregiving amongst male and female peer support workers in two US state prisons. In preparation. 10.1108/IJOPH-08-2025-0062 42545310

[R75] Sullivan Marianne, Ormel Johan, Kempen Gertrudis I. J. M., and Tymstra Tjebbe J.. 1998. Beliefs Concerning Death, Dying, and Hastening Death Among Older, Functionally Impaired Dutch Adults: A One-Year Longitudinal Study. Journal of the American Geriatrics Society 46: 1251–57. 9777907 10.1111/j.1532-5415.1998.tb04541.x

[R76] Templer Donald I. 1971. Death anxiety as related to depression and health of retired persons. Journal of Gerontology 26: 521–23. 4398602 10.1093/geronj/26.4.521

[R77] Toch Hans. 2000. Altruistic activity as correctional treatment. International Journal of Offender Therapy and Comparative Criminology 44: 270–78.

[R78] Tomer Adrian. 1992. Death anxiety in adult life—Theoretical perspectives. Death Studies 16: 475–506.

[R79] Walkey Frank H. 1982. The multidimensional Fear of Death Scale: An independent analysis. Journal of Consulting and Clinical Psychology 50: 466. 7096754 10.1037//0022-006x.50.3.466

[R80] Wang Tingting, Cheung Kam, and Cheng Ho Yu. 2024. Death education interventions for people with advanced diseases and/or their family caregivers: A scoping review. Palliative Medicine 38: 423–46. 38634233 10.1177/02692163241238900

[R81] Wion Rachel K., and Loeb Susan J.. 2016. CE: Original research: End-of-life care behind bars: A systematic review. AJN The American Journal of Nursing 116: 24–36. 10.1097/01.NAJ.0000481277.99686.82 26871892

[R82] Wysokiński Mariusz, Fidecki Wiesław, and Jarosz Mirosław. 2019. Elderly People’s Acceptance of Death: A Study of a Polish Cohort. International Journal of Environmental Research and Public Health 16: 3374. 31547290 10.3390/ijerph16183374 PMC6765774

[R83] Ying Liu, Yuyu Dong, Qinqin Zhang, Yu Yang, Qingxuan Ning, and Zhihuan Zhou. 2024. Death anxiety among caregivers of patients with advanced cancer: A cross-sectional survey. Supportive Care in Cancer 32: 510. 39002026 10.1007/s00520-024-08707-9

[R84] Zarit Steven H., Reever Karen E., and Bach-Peterson Julie. 1980. Relatives of the impaired elderly: Correlates of feelings of burden. The Gerontologist 20: 649–55. 7203086 10.1093/geront/20.6.649

[R85] Zuccala Matteo, Menzies Rachel E., Hunt Caroline J., and Abbott Maree J.. 2022. A systematic review of the psychometric properties of death anxiety self-report measures. Death Studies 46: 257–79. 31809665 10.1080/07481187.2019.1699203

